# Descriptive Investigation of Strongyloidiasis Infection and Characterization of *Strongyloides stercoralis* Using Morphological and Molecular-Based Methods

**DOI:** 10.1155/2020/5431491

**Published:** 2020-08-20

**Authors:** Nayana Gunathilaka, Nilmini Chandrasena, Tharaka Wijerathna, Yoshito Fuji, Deepa Gunasekara, Ruwan Prasanna Gunatilaka, Ranjan Premaratna

**Affiliations:** ^1^Department of Parasitology, Faculty of Medicine, University of Kelaniya, Ragama, Sri Lanka; ^2^Visiting Scholar, Department of Parasitology, Faculty of Medicine, University of Kelaniya, Ragama, Sri Lanka; ^3^Department of Biochemistry and Clinical Chemistry, Faculty of Medicine, University of Kelaniya, Ragama, Sri Lanka; ^4^Department of Medicine, Faculty of Medicine, University of Kelaniya, Ragama, Sri Lanka

## Abstract

Strongyloidiasis is caused by the nematode *Strongyloides stercoralis* which has the unique ability to reproduce and complete its entire life cycle within the human host through its autoinfection cycle. Diagnosis of this infection is important because of its potential to cause fatal hyperinfection syndrome or disseminated infections in those with defective cellular immunity. Parasitological methods based on faecal microscopy and culture often fail to detect low-intensity infections. A multiplex polymerase chain reaction (PCR) assay was developed for the detection of *S*. *stercoralis*, *Ascaris lumbricoides*, and *Enterobius vermicularis* by designing primers specific for the ITS1 region of ribosomal DNA of *S*. *stercoralis* and *A*. *lumbricoides* and 18S region of rRNA of *E*. *vermicularis*. A 61-year-old patient presented with chronic gastrointestinal and respiratory symptoms and weight loss with a stool microscopy positive for helminth larvae. Stool cultures with the Harada–Mori technique yielded L3 larvae which were identified as *S*. *stercoralis* based on morphology. The multiplex PCR performed on DNA extracted from stool elicited the expected band at 129 bp on gel electrophoresis of the PCR yield providing molecular evidence of intestinal strongyloidiasis. The patient's gastrointestinal symptoms improved with a six-day course of albendazole (400 mg twice daily). Negative posttreatment stool microscopy, culture, and PCR confirmed successful clearance of infection. Molecular-based PCR assay is a promising tool to diagnose and assess the therapeutic efficacy of anthelmintics in intestinal helminthiases such as strongyloidiasis.

## 1. Background

Strongyloidiasis is a soil-transmitted helminthiasis (STH), caused by the nematode *Strongyloides stercoralis* and to a lesser extent by the zoonotic species *Strongyloides fuelleborni* and *Strongyloides cf fuelleborni* [1–3]. Considered as the most neglected of the neglected tropical diseases in the world [4], it is endemic in Southeast Asia, Latin America, sub-Saharan America, and parts of Southeast United States [5] affecting an estimated 30–100 million individuals worldwide [6]. The prevalence of infection is on the rise globally, in endemic as well as in nonendemic regions and is underestimated in many countries [7]. In Sri Lanka, strongyloidiasis is infrequently detected in stool surveys compared to other major STH infections [8–10]. However, symptomatic case presentations show a rising trend at present [11–13].

In *S*. *stercoralis* infection, the noninfective rhabditiform larvae that hatch from ovum may rapidly develop into the infective filariform larvae (L3) within the small intestine and cause autoinfection by penetration of either mucus membrane (internal autoinfection) or skin (external autoinfection) or may be excreted to the environment in the noninfective (rhabditiform) stage requiring a soil phase of development to cause new infections. The tropical weather (warm and humid) prevailing in the endemic regions favors the sustenance of parasitic and free-living stages in the soil [3, 14]. Walking barefoot and engaging in soil-related work, poor personal hygiene, unsatisfactory sanitation, and insufficient supplies of drinking water are risk factors for infection. Hence, many resource-poor tropical and subtropical settings provide ideal conditions for transmission [6, 15–17].

The unique ability of *S*. *stercoralis* to replicate within the human host results in infections lasting over several decades [6, 18]. Autoinfection also gives rise to the two potentially fatal complications such as hyperinfection syndrome and disseminated strongyloidiasis seen particularly in individuals with defective cell-mediated immunity.

Uncomplicated intestinal strongyloidiasis is mostly asymptomatic or may present with nonspecific and recurrent symptoms such as diarrhoea, abdominal pain, and urticarial skin rashes or serpiginous skin lesions [6, 19]. The recurrent nature of symptoms and a high peripheral eosinophil count would direct clinical suspicion towards strongyloidiasis. However, establishing a definitive diagnosis by parasitological methods may be challenging as larvae are excreted intermittently and in low quantities. Thus, the sensitivity of a single stool smear examination is only about 50% or even less [20, 21]. Baermann concentration methods and coprocultures increase the sensitivity but are cumbersome, time-consuming, and difficult to use in large-scale surveys [22].

Serology-based antigen or antibody detection assays have the potential to be used in the diagnosis of strongyloidiasis and other intestinal helminthiases due to their high sensitivity [7]. These include the enzyme-linked immunosorbent assay (ELISA) and its derived procedures such as Falcon assay screening test ELISA, dot-ELISA, luciferase immunoprecipitation system (LIPS), immunoblotting, indirect immunofluorescent antibody test (IFAT)/direct immunofluorescent antibody test, and rapid diagnostic tests (RDTs) [23]. However, drawback associated with the usage of antibody-based techniques is their low specificity due to cross-reactivity with other helminthiases, and the persistent nature of antibodies limits their use in areas endemic for strongyloidiasis as they cannot distinguish active infections from the past infections [24–26]. Furthermore, the sensitivity may be lower in severely immunocompromised patients. The invasive nature of serology-based tests is a also disadvantage compared to faecal-based methods [23].

Polymerase chain reaction- (PCR-) based applications are considered a priority area in the field of modern diagnostics. It has shown a higher sensitivity than microscopy, particularly at low intensities of infections [25]. Although molecular-based diagnostics are infrequently applied in the diagnosis of intestinal helminthiases, they are considered a necessity in the STH elimination drive to detect areas with low intensities of infection [27, 28]. Sri Lanka has documented great strides in the control of intestinal helminthiases reaching national prevalence rates of less than 1% [10]. Thus, in keeping with the global recommendation, it is timely to venture into molecular diagnostics to detect intestinal parasitic infections. Therefore, the present study illustrates the first successful attempt of molecular-based detection of *S*. *stercoralis* in Sri Lanka.

The present investigation was conducted in March 2018 based on a patient referred to the Colombo North Teaching Hospital of Sri Lanka, for management of symptomatic intestinal strongyloidiasis. The patient and the faecal samples were referred to the Department of Parasitology, Faculty of Medicine, University of Kelaniya, Sri Lanka, for diagnostic confirmation and posttreatment follow-up.

## 2. Case Presentation

A 61-year-old farmer was admitted to the CNTH with colicky right epigastric pain, generalized abdominal discomfort, 8–10 times of watery diarrhoea, and urge incontinence for six days' duration. He had no blood or mucus in the stool. The symptoms were prominent after meals. His appetite was poor and had a significant weight loss (about 21 kg) over the past two months. He also had a cough for about three months' duration which was associated with palpitations and faintishness. He was on treatment for essential hypertension, ischemic heart disease, and chronic kidney disease. A three-day course of albendazole 400 mg per day had been administered prior to current admission based on a presumptive diagnosis of intestinal helminthiasis but symptoms persisted.

On examination, he was pale and wasted. His left cervical nodes were enlarged. There were no skin rashes. Examination of the cardiovascular and respiratory systems was normal. There was tenderness in the right epigastrium. His total leucocyte count was 9.8 × 10^3^ *µ*l with 25% eosinophils, 245 × 10^3^ *µ*l of platelets, and 9.7 g/dl of haemoglobin. His blood picture indicated a severe iron deficiency anaemia and peripheral eosinophelia. The erythrocyte sedimentation rate was 27 mm/1st hour, and C-reactive protein was 15.1 mg/dl. His serum creatinine was 1.4 mg/dl, and blood urea and serum electrolytes were within the normal range. His protein profile was low with a total serum protein of 5.1 g/dl with albumin and globulins of 2.9 g/dl and 2.2 g/dl, respectively. Serum transaminases were within the normal range.

Ultrasound scan of the abdomen was normal, and chest X-ray indicated a globular heart. Faecal and sputum specimens were obtained a week following the initial course of anthelmintics for parasitological and molecular analysis at the Department of Parasitology.

### 2.1. Parasitological Examination

**Figure 1:**
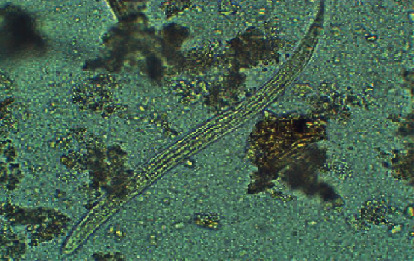
L1 rhabditiform larvae of *Strongyloides stercoralis* in direct faecal smear.

**Figure 2:**
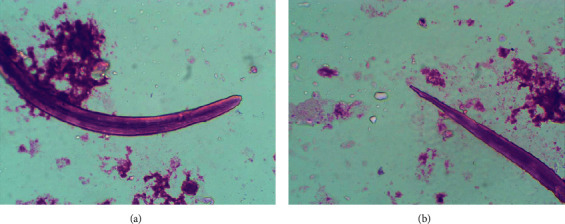
L3 filariform larvae of *Strongyloides stercoralis* isolated from the Harada–Mori culture showing (a) long esophagus and (b) tail with a notched tip.

Direct faecal microscopy was positive for rhabditiform larvae (average length of 210–248 *µ*m) which were identified presumptively as those of *S*. *stercoralis* by their short buccal capsule and prominent genital primordium (Figure 1). There were no other parasites (helminth larvae, ova, or cysts) in the faecal sample. Sputum microscopy was negative for helminth larvae. Coprocultures using the Harada–Mori filter paper technique yielded L3 filariform larvae on day seven which were identified as those of *S*. *stercoralis* by their long esophagus and tail with a notched tip (Figure 2). A second course of albendazole (400 mg twice daily) for six days was administered, and faecal samples obtained a week after the second anthelmitics course were negative for rhabditiform larvae on direct microscopy. His abdominal symptoms improved and bowel habits became normal.

## 3. Molecular Confirmation

### 3.1. DNA Extraction

Genomic DNA was extracted directly from faecal samples (pre- and posttreatment) and from the Harada–Mori coproculture yield using QIAamp DNA Stool Mini Kit (QIAGEN, Germany) according to the manufacturer's protocol.

### 3.2. Primer Designing

Specific primers for *Ascaris lumbricoides*, *Enterobius vermicularis*, and *S*. *stercoralis* were designed based on the polymorphic sites of ITS1, 18S rRNA, and ITS2 by Primer Premier 5.0 and combined with the previously published specific primers for *S*. *stercoralis* [29] to generate target fragments of different sizes. The three pairs of specific primers shared similar annealing temperatures and were predicted to form no secondary structures or primer-dimers; their sequences and expected sizes of amplicons are shown in Table 1. The relative alignments of primers are included as supplementary material. (*available*here)

### 3.3. Simplex PCR Assays for Each Target

PCR assays for each target species were performed initially with each pair of primers. All reactions were performed in 20 *μ*L volume containing 1.2 *μ*l primer mix (0.3 *μ*M each), 10.0 *μ*l HotStarTaq Plus Master Mix 2x (Promega, USA), 2.0 *μ*l CoralLoad concentrate 10x (Promega, USA), 5.8 *μ*l PCR water, and 1.0 *μ*l of DNA template. The PCRs were programmed as 95°C for 5 min, followed by 40 cycles of 95°C for 30 s, 58°C for 30 s, 72°C for 30 s, and a final extension step at 72°C for 5 min. Negative controls with sterile distilled water and DNA extracted from negative stool were run at each trial separately.

### 3.4. Multiplex PCR Assay

A multiplex PCR assay was performed using 20.0 *μ*l of solution containing 1.2 *μ*l primer mix (0.3 *μ*M each), 10.0 *μ*l HotStarTaq Plus Master Mix 2x (Promega, USA), 2.0 *μ*l CoralLoad concentrate 10x (Promega, USA), 5.8 *μ*l PCR water, and 1.0 *μ*l of DNA template. The cycling conditions were hot start at 95°C for 5 min followed by 40 cycles each of denaturation at 95°C for 30 s, annealing at 58°C for 30°s, and extension at 72°C for 28°s followed by a final extension at 72°C for 5 minutes. The samples were finally cooled at 4°C for 5 minutes.

### 3.5. Agarose Gel Electrophoresis and Interpretation of Results

**Figure 3:**
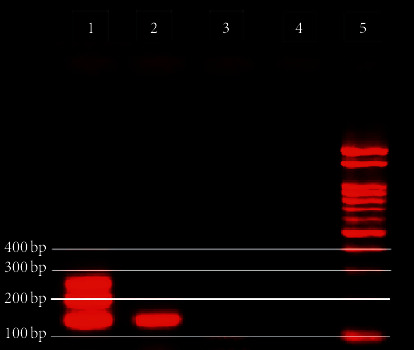
Multiplex PCR assay to differentiate *A*. *lumbricoides*, *E*. *vermicularis*, and *S*. *stercoralis*. Lane 1: positive control for three targets; lane 2: *S*. *stercoralis* in stool sample; lane 3: DNA extracted from negative stool; lane 4: negative control; lane 5: 100 bp ladder (Promega).

The PCR products were loaded on a 2.5% agarose gel stained with ethidium bromide (0.5 *μ*g/ml) with a 100 bp molecular weight marker (Promega lambda). The PCR products were separated by electrophoresis (100 mV for 30 min) and visualized using a UV Transilluminator (Maestrogen, Taiwan, and ImageJ software package NIH, USA). The pretreatment faecal specimen elicited the expected band at the region of 129 bp for *S*. *stercoralis* (Figure 3). Molecular analysis of posttreatment faecal samples failed to produce a band.

## 4. Discussion

Intestinal strongyloidiasis is the most evasive and underdiagnosed infection of all helminth infections. However, a delay in diagnosis could be fatal in situations of therapeutic immunodepression, debilitating disease, and other conditions with altered immune response [30]. Despite Sri Lanka being a tropical country with favorable climatic conditions for the sustenance of the life cycle of *S*. *stercoralis*, the infection rates are very low (0–1.6%) [8–10] compared to neighboring countries such as India (6.6–12.2%) [6]. It is possible that these rates are an underestimate as the survey methods employed were relatively insensitive for strongyloidiasis (single direct stool smears and Kato–Katz) [20]. Isolated cases of infection being reported among immunocompromised as well as immunocompetent patients provide evidence of its presence in Sri Lanka [11–13]. This patient being a paddy cultivator from a rural area with probable deficient sanitation may have exposed him to infection.

Therapeutic management of strongyloidiasis is not very satisfactory. Ivermectin is considered the first-line therapy for chronic strongyloidiasis [31] as it is more effective and better tolerated than thiabendazole and albendazole, respectively [32–34]. Since ivermectin is not registered for human use in Sri Lanka, a high-dose regimen of albendazole (400 mg twice/day) administered for six days successfully eliminated the infection. The patient was followed up with posttreatment stool examinations to ensure clearance of infection as treatment failures and relapses after antiparasitic therapy is frequently noted [35, 36].

Molecular-based assays on stool DNA samples are increasingly being used for diagnosis of intestinal helminth infections including strongyloidiasis with increased detection rates compared to parasitological techniques [7]. Routine parasitological techniques lack sensitivity; serology lacks specificity as cross-reacting antibodies do not distinguish past and present infections, and the sensitivity is lower in the immunosuppressed [37]. Endoscopic observations may be nonspecific ranging from normal mucosal appearance to severe duodenitis or colitis [38, 39]. Thus, there is an urgent requirement for molecular-based diagnostics for strongyloidiasis. Although molecular assays have been established and validated for the diagnosis of strongyloidiasis in other countries [3, 29], no such published records are available for Sri Lanka. This is the first report of the successful application of a molecular-based diagnostic for intestinal helminthiases in Sri Lanka using a multiplex PCR, facilitating the detection of multiple infections within a single platform with probable greater sensitivity than the traditional methods.

In designing the primers for the multiplex PCR, two conserved regions, namely, ITS1 and 18S rRNA were utilized to clearly discriminate the bands (molecular weight) elicited by the PCR products. Although diagnostic assays based on multiplex PCR are in use, they are either quantitative or combined with DNA probes. Application of such assays to routine diagnostic services may not be cost-effective in resource-poor settings such as Sri Lanka. Designing the primers to yield a PCR product of unique size for each targeted species enabled the performance of the multiplex PCR within the conventional PCR system. The 18S ribosomal RNA gene used frequently for pathogen-specific detection and the internal transcribed spacer 1 (ITS1) which is more species specific were selected for this assay.

The major limitation of implementing molecular-based tests in routine diagnosis is its associated high cost (need for well-equipped laboratories and expertise) compared with traditional methods. However, a price comparison of a multiplex qPCR platform for STH was estimated to cost about the same in consumables as that of microscopy in Malaysia [40]. The price estimate of the current test (material processing and multiplex PCR) was Sri Lankan Rupees 650 (USD 3.6) per test, indicating that the cost may not be a prohibitive factor for its application in required settings.

Application of serological tests would have complemented the diagnostic findings in the present case scenario but would not have been a reliable indicator of clearance of infection due to the test limitations stated above. The present study did not focus on the serological testing due to nonavailability of tests and lack of funds for experimental development of tests. On the other hand, the use of such technique for individual diagnosis would be costly for a developing country like Sri Lanka. Therefore, multiplex PCR aiming different targets would be beneficial, low cost, and more appropriate.

The recent reforms in the health sector of Sri Lanka, occurring parallel to the socioeconomic growth, has increased the availability of sophisticated medicines and techniques that include immunomodulatory drugs and organ transplant. As a consequence, there is an expansion in the immunocompromised population who are vulnerable to hyperinfection or disseminated strongyloidiasis. Therefore, a sensitive diagnostic tool is required to screen for strongyloidiasis prior to such interventions. This molecular-based diagnostic may be applicable in screening for strongyloidiasis in such situations. Such a tool could also be deployed for surveillance and disease mapping of other coendemic intestinal helminthiases particularly those which evade detection in stool samples such as enterobiasis enabling estimation of infection rates among adolescent and adult populations.

## 5. Conclusions

The present report documents the development of a multiplex PCR for the diagnosis of three intestinal helminth infections such as *S*. *stercoralis*, *A*. *lumbricoides*, and *E*. *vermicularis* in Sri Lanka. This tool was used in parallel with parasitological techniques for establishing the diagnosis of a symptomatic case of intestinal strongyloidiasis with promising evidence of its applicability as a diagnostic tool and a tool to assess the therapeutic response of anthelmintics among individuals and communities.

## Figures and Tables

**Table 1:** The Primers designed for molecular assay with expected band sizes.

Species	Target gene	Primer sequence (5′ to 3′)	Expected band size (bp)
*A*. *lumbricoides*	ITS1	F: GTGATGTAATAGCAGTCGGCGGTT	242
*E*. *vermicularis*		R: CTACTCGACTCGAAACGAGGAGCTT F: GGTGCTGAACTAAGTACATCTCAGTGT	
	18S	R: GGAAACCAACAAAATAGACCCGTAATCGTATTC F: ATCGTGTCGGTGGATCATTCGGTT	187
*S*. *stercoralis*	ITS1	R: AATAGTATAAAATACTATTAGCGCCATTTGCATTC	129

## Data Availability

All data are available with the authors and will be provided upon reasonable request.

## Abbreviations

ELISA: Enzyme-linked immunosorbent assay
CNTH: Colombo North Teaching Hospital
IFAT: Indirect immunofluorescent antibody test
LIPS: Luciferase immunoprecipitation system
PCR: Polymerase chain reaction
RDT: Rapid diagnostic test
STH: Soil-transmitted helminthiasis.

## Supplementary Materials

Supplementary Materials Supplementary 1: relative alignments of primers designed for (a) *Ascaris lumbricoides*, (b) *Strongyloides stercoralis*, and (c) *Enterobius vermicularis*.
