# Supplementary material for: Descriptive Investigation of Strongyloidiasis Infection and Characterization of Strongyloides stercoralis Using Morphological and Molecular-Based Methods
Source: Case Rep Infect Dis. 2020 Aug 20;2020:5431491. doi: 10.1155/2020/5431491 (PMC7455842; doi:10.1155/2020/5431491)
Supplement: Supplementary Materials — Supplementary 1: relative alignments of primers designed for (a) Ascaris lumbricoides, (b) Strongyloides stercoralis, and (c) Enterobius vermicularis. [file 5431491.f1.docx]

**Supplimentary material 1**


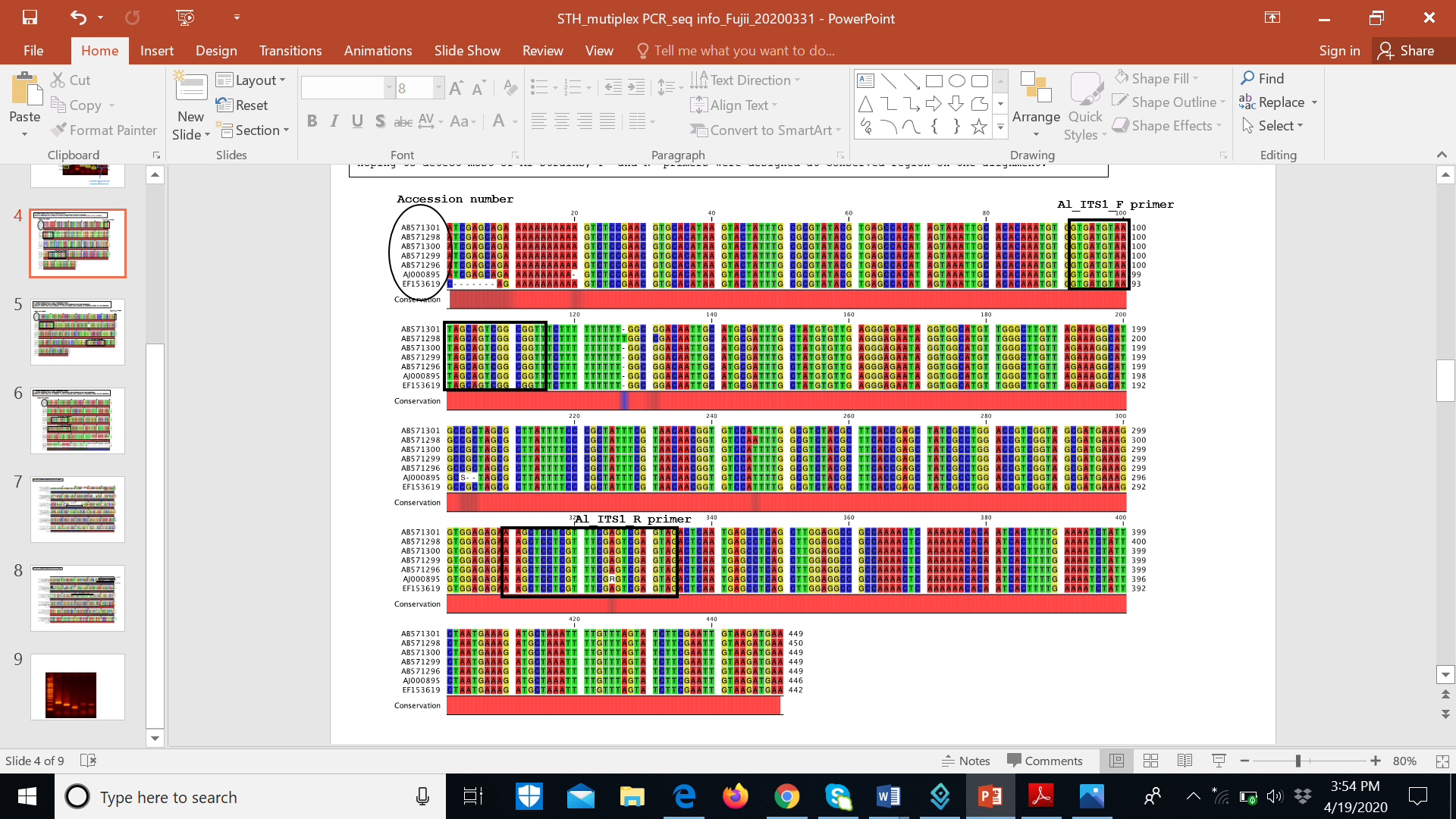


**1a.** Relative allignment of primers designed for *Ascaris lumbricoides.*


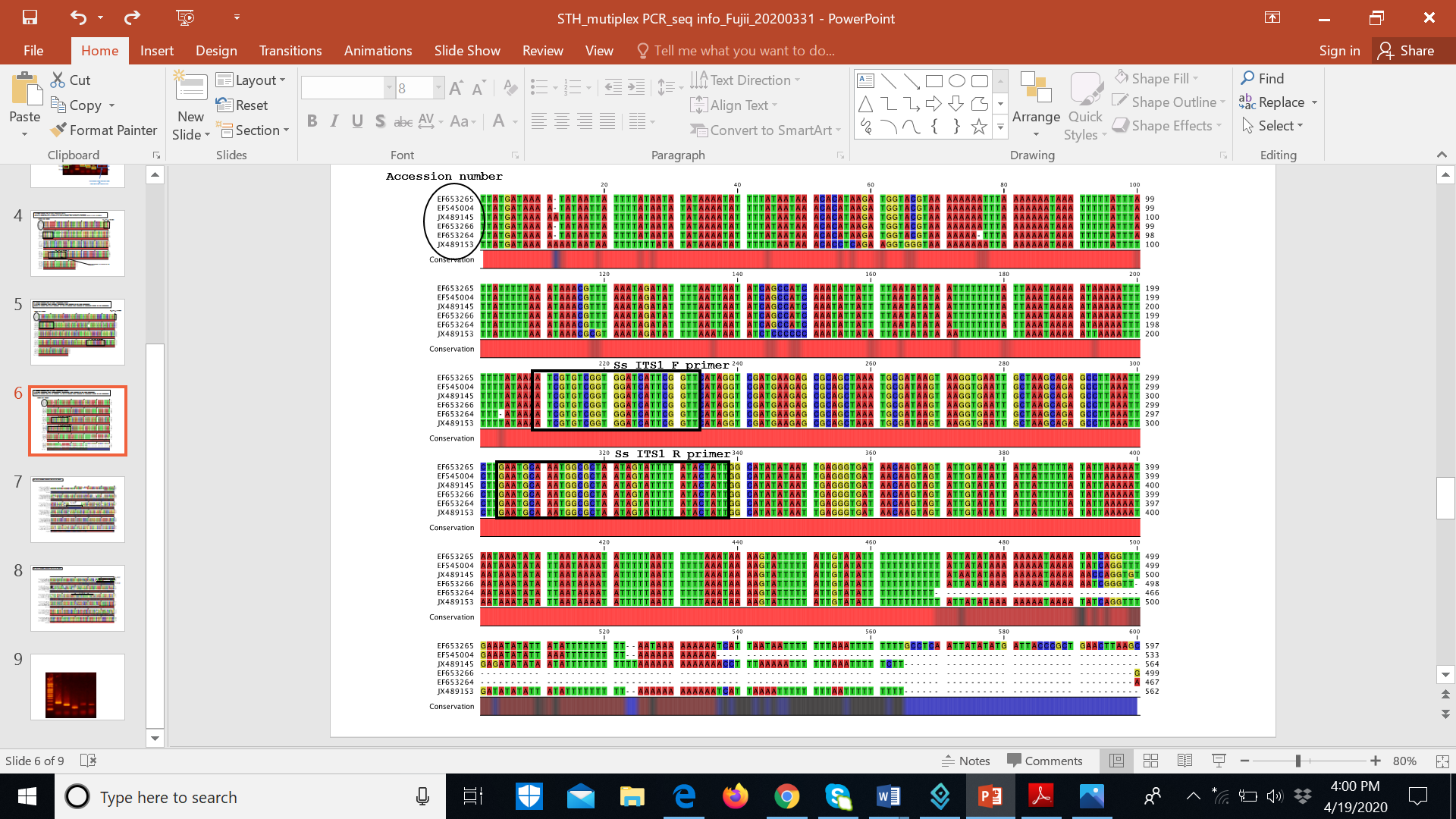


**1b.** Relative allignment of primers designed for *Strongyloides stercoralis*


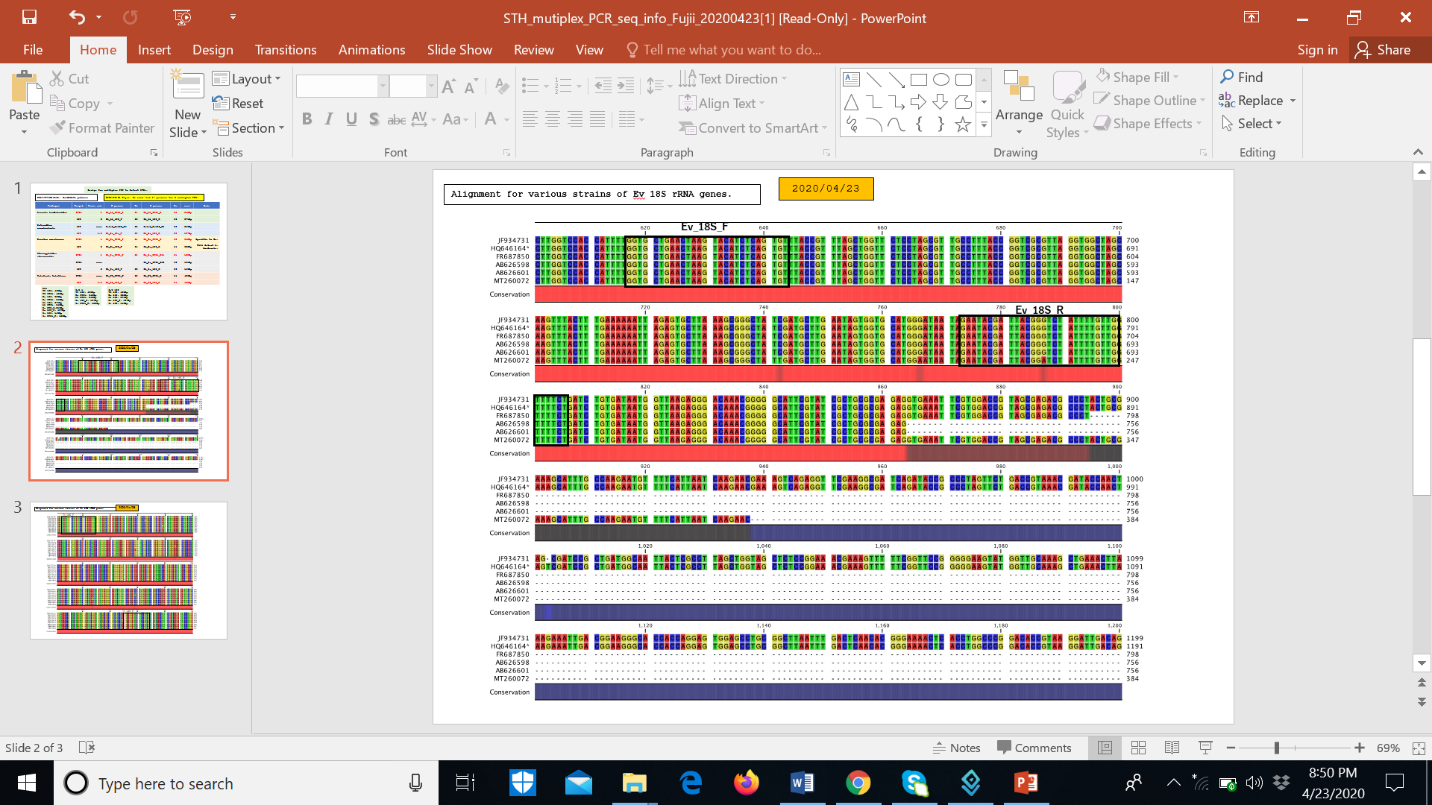


**1c.** Relative allignment of primers designed for *Enterobius vermicularis*
